# T-regulatory cell treatment prevents chronic rejection of heart allografts in a murine mixed chimerism model

**DOI:** 10.1016/j.healun.2013.11.004

**Published:** 2014-04

**Authors:** Nina Pilat, Andreas M. Farkas, Benedikt Mahr, Christoph Schwarz, Lukas Unger, Karin Hock, Rupert Oberhuber, Klaus Aumayr, Fritz Wrba, Thomas Wekerle

**Affiliations:** a Division of Transplantation, Department of Surgery, Medical University of Vienna, Vienna; b Department of Visceral, Transplant, and Thoracic Surgery, Center of Operative Medicine, Innsbruck Medical University, Innsbruck; c Institute of Clinical Pathology, Medical University of Vienna, Vienna, Austria

**Keywords:** mixed chimerism, tolerance, regulatory T cells, chronic rejection, heart transplantation, costimulation blockade

## Abstract

**Background:**

The mixed chimerism approach induces donor-specific tolerance in both pre-clinical models and clinical pilot trials. However, chronic rejection of heart allografts and acute rejection of skin allografts were observed in some chimeric animals despite persistent hematopoietic chimerism and tolerance toward donor antigens in vitro. We tested whether additional cell therapy with regulatory T cells (Tregs) is able to induce full immunologic tolerance and prevent chronic rejection.

**Methods:**

We recently developed a murine “Treg bone marrow (BM) transplantation (BMT) protocol” that is devoid of cytoreductive recipient pre-treatment. The protocol consists of a moderate dose of fully mismatched allogeneic donor BM under costimulation blockade, together with polyclonal recipient Tregs and rapamycin. Control groups received BMT under non-myeloablative irradiation and costimulation blockade without Treg therapy. Multilineage chimerism was followed by flow cytometry, and tolerance was assessed by donor-specific skin and heart allografts.

**Results:**

Durable multilineage chimerism and long-term donor skin and heart allograft survival were successfully achieved with both protocols. Notably, histologic examination of heart allografts at the end of follow-up revealed that chronic rejection is prevented only in chimeras induced with the Treg protocol.

**Conclusions:**

In a mouse model of mixed chimerism, additional Treg treatment at the time of BMT prevents chronic rejection of heart allografts. As the Treg-chimerism protocol also obviates the need for cytoreductive recipient treatment it improves both efficacy and safety over previous non-myeloablative mixed chimerism regimens. These results may significantly impact the development of protocols for tolerance induction in cardiac transplantation.

Deliberate induction of donor-specific tolerance is a major goal in transplantation medicine as it would eliminate the need for chronic immunosuppression and the risk of chronic rejection. The mixed chimerism approach was shown to be a successful tolerance strategy in experimental animal models^1^ and in the clinical setting.^2^, ^3^, ^4^ However, clinical application of this approach is hindered by the toxicity of available bone marrow transplantation (BMT) protocols.

Intensive research has led to the development of experimental BMT protocols devoid of global T-cell depletion.^5^ Costimulation blockade-based BMT protocols seem to be superior to protocols that include T-cell depletion as they are considered to be less cytotoxic. Furthermore, underlying tolerance mechanisms may differ between these approaches, as tolerance is established soon after BMT before peripheral deletion is complete, suggesting an important role for non-deletional tolerance mechanisms in costimulation blockade-based models. It was demonstrated that (host) regulatory T cells (Tregs) are crucial in the induction phase of tolerance but have no critical role in tolerance maintenance^6^ in a costimulation blockade-based BMT model employing non-myeloablative doses of total body irradiation (TBI). More recently, we showed that therapeutic administration of Tregs leads to mixed chimerism and donor-specific tolerance in a costimulation blockade-based BMT protocol devoid of irradiation or cytotoxic drugs, underlining the importance as well as potency of Tregs in this model.^7^ In this “Treg BMT protocol,” tolerance seems to be mediated mainly by non-deletional mechanisms, as it was shown that levels of chimerism remained rather low and deletion of donor-reactive T cells was much less pronounced.

In experimental models, especially mice, skin grafts are considered to be the most stringent test for tolerance and skin graft survival is usually followed for >100 days, which is commonly considered to indicate permanent survival. Nevertheless, although skin grafts may remain macroscopically intact for >100 days, and histologic examination does not reveal major changes in skin architecture, the risk of chronic rejection cannot be fully assessed as skin grafts are only secondarily vascularized with endothelium derived from the recipient. Thus, permanent survival of skin allografts in mixed chimeras does not allow for a reliable conclusion as to whether full donor-specific tolerance capable of preventing chronic rejection of solid-organ allografts has been established. This is of particular interest, as some studies demonstrated chronic rejection of primarily vascularized heart grafts in skin graft–tolerant recipients.^8^, ^9^ This state, called “split tolerance,” is an immunologic phenomenon that is still incompletely understood.^10^

In this study we examined the potency of Tregs in preventing chronic rejection of primarily vascularized heart allografts in a murine BMT model without cytotoxic recipient pre-conditioning.

## Methods

### Animals

Female C57BL/6 (B6, recipient, *H-2*^*b*^), BALB/c (donor, *H-2*^*d*^) and C3H/HeNCrl (C3H, third party, *H-2*^*k*^) mice were purchased from Charles River Laboratories (Sulzfeld, Germany). The animals were housed under specific pathogen-free conditions and were used at 6 to 12 weeks of age. All experiments were approved by the local review board of the Medical University of Vienna and were performed in accordance with national and international guidelines of laboratory animal care. All mice received humane care in compliance with the “Principles of Laboratory Animal Care,” formulated by the National Society for Medical Research, and *The Guide for the Care and Use of Laboratory Animals*, prepared by the Institute of Laboratory Animal Resources and published by the National Institutes of Health (NIH Publication No. 86-23, revised 1996).

### BMT protocols

Groups of age-matched B6 recipients received approximately 2 × 10^7^ unseparated BM cells recovered from BALB/c donors (intravenous injections at Day 0) and costimulation blockade with cytotoxic T-lymphocyte–associated antigen 4 immunoglobulin (CTLA4Ig; 0.5 mg, Day 2) and anti-CD154 monoclonal antibodies (MAb; MR1; 1 mg, Day 0). Groups of mice received additional Treg treatment (described in what follows) and a short course of rapamycin (0.1 mg/mouse, Days −1, 0 and 2).^7^ Alternatively, BMT recipients received 3-Gy TBI prior to BMT (Day −1).^5^ Anti-CD154 MAb was purchased from BioXCell (West Lebanon, NH), rapamycin (sirolimus) was purchased from LD Laboratories (Woburn, MA), human CTLA4Ig (abatacept) was generously provided by Bristol-Myers, Squibb Pharmaceuticals (Princeton, NJ).

### Generation of Tregs

Induced Tregs (iTregs) were generated as described previously.^7^ Briefly, CD4^+^ cells were isolated from B6 spleen and lymph nodes by magnetic bead sorting (L3T4 Microbeads, Miltenyi Biotec), and cultivated in plates coated with 10 µg/ml anti-CD3 (145-2C11), 1 µg/ml anti-CD28 (37.51) (BD Pharmingen) in the presence of 100 U/ml interleukin-2 (IL-2; Sigma) and 5 ng/ml recombinant human transforming growth factor-beta (rhTGF-β; R&D Systems) for 5 days. At the end of culture, the Treg-enriched cell population was used for therapeutic administration without additional sorting steps. FoxP3 expression was usually >80%. Then 3 × 10^6^ cells were injected intravenously per mouse.

### Heart transplantation

Cervical heterotopic heart transplantation was performed 6 to 8 weeks after BMT with a modified cuff technique for revascularization, as described previously.^11^ Briefly, after preparation of the right external jugular vein and common carotid artery cuffs, the donor heart was harvested and transferred to the neck of the recipient. Main pulmonary artery and aorta were anastomosed to the recipient’s external jugular vein and carotid artery, respectively. Cardiac allograft survival was determined by daily palpation and observation under the microscope with complete cessation of heart beats indicating end of graft survival (confirmed by histopathologic analysis).

### Skin grafting

Full-thickness tail skin from donor (BALB/c) and fully mismatched third-party (C3H) mice was grafted 4 to 6 weeks after BMT and visually inspected thereafter at short intervals. Grafts were considered to be rejected when <10% remained viable.

### Anti-donor antibodies

Recipient serum harvested >3 months post-BMT was heat-inactivated and incubated with recipient-type, donor-type and third-party-type thymocytes (which are low in Fc receptors, reducing background staining). Binding of serum IgG antibodies to thymocytes was analyzed by flow cytometry using fluorescein isothiocyanate (FITC)-conjugated rat anti-mouse IgG1 and IgG2a/2b (BD Pharmingen).

### Mixed lymphocyte reaction

Mixed lymphocyte reactions (MLRs) were performed as described in detail previously.^12^ Briefly, 4 × 10^5^ responder splenocytes were incubated in triplicate with 4 × 10^5^ irradiated (at 30 Gy) stimulator cells of either B6 (recipient), BALB/c (donor) or C3H (third party) origin or with medium only. After 72 hours of incubation, cells were pulsed with [^3^H]-thymidine (Amersham Biosciences, UK) for 18 hours. Incorporated radioactivity was measured using scintillation fluid in a β-counter. Stimulation indices (SIs) were calculated in relation to medium controls. Results represent averaged data from each group.

### Antibodies and flow-cytometric analysis

Multicolor flow-cytometric analysis of multilineage macrochimerism and Vβ-subunit expression were performed as described previously.^5^, ^13^ Chimerism was calculated as the net percentage of donor MHC Class I^+^ (*H-2D*^*d*^, 34-2-12) cells among specific leukocyte lineages, as described previously.^5^, ^13^ Mice were considered chimeric if donor cells were detectable by flow cytometry within both the myeloid lineage and at least one lymphoid lineage. For analysis of Treg phenotype, MAbs with specificity against CD4 (RM4-4) and CD25 (7D4) were used. For intracellular staining, a FoxP3 (FJK-16s) staining kit (eBioscience) was used according to the manufacturer’s protocol. Propidium iodide (PI) was used for dead cell exclusion when appropriate. Surface staining was performed according to standard procedures and flow-cytometric analysis was done on a Coulter Cytomics FC 500 System using CXP software (Coulter, Austria) for acquisition and analysis.

### Histologic analysis

Four-micron sections were cut from paraffin-embedded tissue fixed in 4.5% formalin (with buffered pH 7.5), stained with hematoxylin–eosin (HE), Giemsa–elastica van Gieson (EvG) stain according to standard protocols, and analyzed by an experienced clinical pathologist in a blinded fashion. Heart allografts were scored according to the International Society for Heart and Lung Transplantation (ISHLT) 2005 guidelines for cellular rejection (Grade 0—no rejection; Grade 1R–mild, interstitial and/or perivascular infiltrate with up to 1 focus of myocyte damage; Grade 2R—moderate, 2 or more foci of infiltrate with associated myocyte damage; Grade 3R—severe, diffuse infiltrate with multifocal myocyte damage with or without edema, hemorrhage and vasculitis).^14^

### Statistics

A 2-sided Student's *t*-test with unequal variances was used to compare percentages of Vβ-family–positive cells, chimerism levels and SI values between groups. Fisher’s exact test was used to compare chimerism rates between groups and rejection scores. Skin and heart allograft survival were calculated according to the Kaplan–Meier product limit method and compared between groups using the log-rank test. *p* < 0.05 was considered statistically significant.

## Results

### Generation of TGF-β–induced Tregs

In previous work we reported that therapeutic administration of polyclonal recipient Tregs enhances BM engraftment and therefore allows reduction of recipient pre-conditioning.^7^, ^15^ We investigated the potency of different techniques for Treg generation (retroviral transduction with Forkhead box P3 [FoxP3-Tregs], in vitro activation of natural CD4^+^CD25^+^ Tregs [nTregs] and TGF-β induction [iTregs]^7^) to find the most suitable Treg population for use in the mixed chimerism approach. All tested Treg populations demonstrated similar suppressive potency in vitro and in vivo.^7^ In the experimental setting, iTregs have advantages over other Treg populations. They are relatively easy to obtain in large numbers, whereas nTregs are low in number and cumbersome to expand in vitro. Moreover, compared with FoxP3-Tregs, there are no safety concerns regarding insertional mutagenesis secondary to gene insertion into the host chromosome, an event that could lead to disruption or activation of cellular genes. Importantly, in this specific model, immunosuppressive potency of Tregs is only needed temporarily for prevention of BM rejection by costimulation blockade-resistant alloreactive cells and induction of immunoregulatory pathways within the BM recipient.^7^, ^15^ In contrast to thymus-derived nTregs, iTregs have also been reported to have a similar T-cell receptor (TCR) repertoire as conventional T cells, which also includes a higher percentage of TCR with specificity against alloantigens.^16^

**Figure 1:**
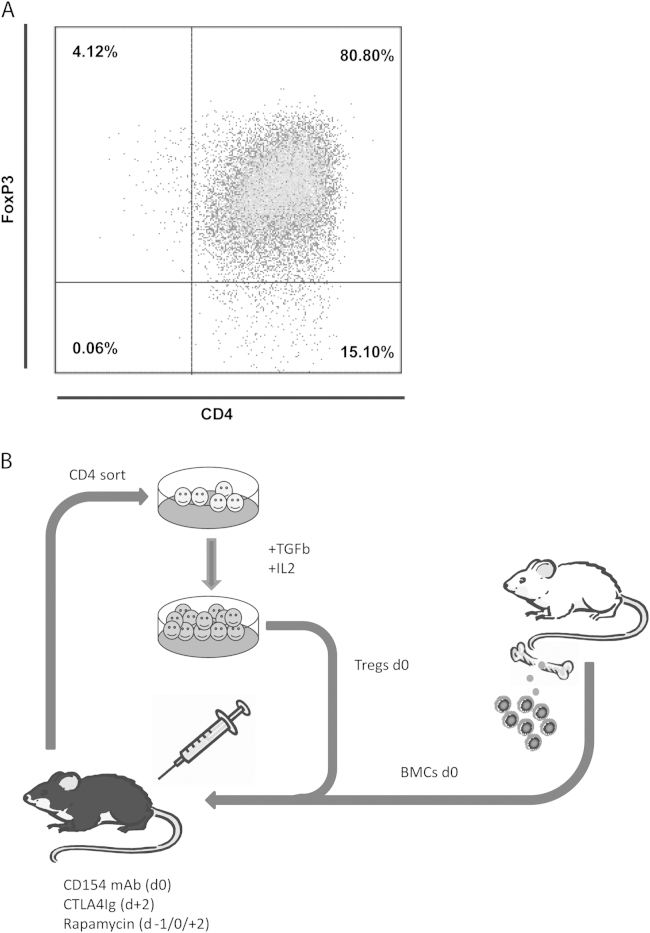
Efficient expression of FoxP3 in TGF-β–induced Tregs enhances BM engrafment in a murine mixed chimerism model. (A) Representative FACS blot depicting FoxP3 expression among CD4 T cells after in vitro cultivation in the presence of TGF-β. (B) Schematic drawing of the non-cytotoxic BMT protocol using Tregs. Recipient-type CD4 T cells were separated by magnetic bead sorting and cultivated in the presence of TGF-β in vitro. Tregs were infused with fully mismatched allogeneic donor BM under the cover of costimulation blockade and rapamycin.

The percentage of FoxP3^+^ cells after 5 days in vitro culture was usually around 80% (Figure 1A). Cells were used without any further sorting at a dose of 3 × 10^6^ cells per recipient (which corresponds to ~2.4 × 10^6^ FoxP3^+^ CD4 cells/mouse [120 × 10^6^ Tregs/kg]). Tregs were administered simultaneously with allogeneic BM to allow activation of alloreactive Tregs, potentially enhancing their suppressor activity (Figure 1B).

### Therapeutic administration of in vitro–induced polyclonal Tregs leads to low but persistent levels of multilineage chimerism

**Figure 2:**
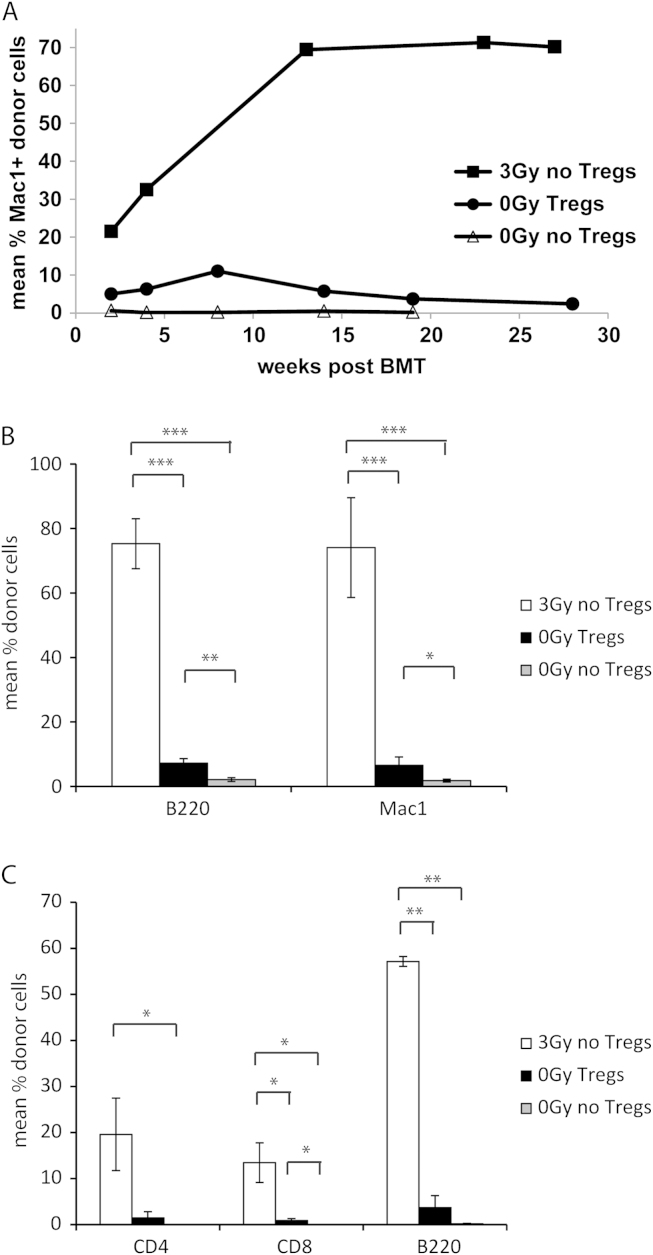
Treg treatment induces low but persistent levels of hematopoietic chimerism without the need for recipient irradiation. (a) Groups of B6 mice received fully mismatched BALB/c BM cells (20 × 10^6^), combined costimulation blockade with CTLA4Ig and MR1, and either pre-treatment with 3-Gy TBI (3 Gy, 6/6 chimeras), polyclonal recipient-type Tregs with short-term rapymycin (5/5 chimeras) or rapaymycin alone (0/5 chimeras). Long-term donor (*H-2D*^*d*^) chimerism among leukocytes of the myeloid (Mac1^+^) lineage was assessed by flow-cytometric analysis of peripheral blood at multiple time-points. (B, C) Multilineage chimerism in lymphoid tissue (BM, spleen) was assessed at the end of follow-up (~7 months post-BMT). (B) Mean levels of B-cell and myloid chimerism within BM are shown for irradiated BMT recipients (3 Gy, open bars, *n* = 6), Treg-treated BMT recipients (0-Gy Tregs, filled bars, *n* = 4) and the unirradiated control group (0-Gy control, shaded bars, *n* = 5). (C) Mean levels of CD4 T-cell, CD8 T-cell and B-cell chimerism among splenocytes are shown for irradiated BMT recipients (3 Gy, open bars, *n* = 6), Treg-treated BMT recipients (0-Gy Tregs, filled bars, *n* = 4) and the unirradiated control group (0-Gy control, shaded bars, *n* = 5). ^***^p < 0.0005, ^**^p < 0.005 and ^*^p < 0.05 (Student’s *t*-test). Error bars indicate standard deviation. Data represent multiple experiments.

Combined treatment with Tregs, costimulation blockade (anti-CD154 MAb at Day 0: 1 mg/mouse; CTLA4Ig at Day 2: 0.5 mg/mouse) and rapamycin (Days −1, 0 and 2: 0.1 mg/mouse) led to the engraftment of a conventional number of BALB/c BM cells in otherwise untreated wild-type B6 recipients (5 of 5 chimeras in 0-Gy Tregs vs 0 of 5 chimeras in 0-Gy control; *p* = 0.008). Although this protocol reliably induced long-term chimerism, the chimerism levels were substantially lower than in the group employing 3-Gy TBI (e.g., myeloid chimerism 5.75% 0-Gy Tregs vs 69.73% 3 Gy [*p* < 0.0001] at 3 months post-BMT; 2.39% 0-Gy Tregs vs 70.17% 3-Gy [*p* < 0.0001] at 7 months post-BMT) (Figure 2A). Notably, chimerism in the Treg-treated group was of a multilineage nature and chimerism levels in peripheral blood correlated with chimerism in lymphoid organs (BM and spleen, 7 months post-BMT; Figure 2B and C). Levels of T-cell chimerism, generally thought to correlate with successful tolerance induction^17^, ^18^—albeit present—remained low in all tested tissues. Multilineage chimerism persisted and remained stable for the length of follow-up (up to 7 months post-BMT), suggesting successful engraftment and survival of donor hematopoietic stem cells in chimeras of both groups.

### Treg treatment induces donor-specific tolerance and prevents chronic rejection in heart allografts

**Figure 3:**
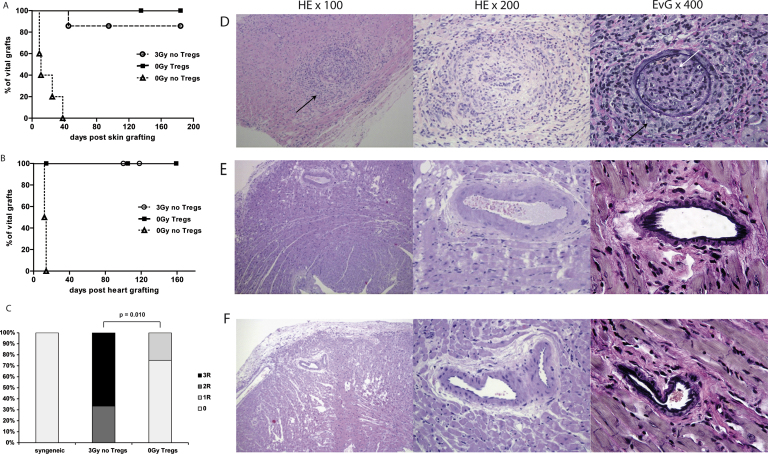
Treg treatment induces full donor-specific tolerance and prevents chronic rejection. (A) Tolerance was assessed by grafting fully mismatched skin allografts. Donor-specific BALB/c skin survived indefinitely in most chimeras induced by irradiation (3 Gy, *n* = 7, open circles) and all chimeras treated with Tregs (0-Gy Tregs, *n* = 5, filled squares), whereas control mice uniformly rejected donor-specific grafts (0-Gy control, *n* = 5, open triangles). (B) Cardiac allograft survival was monitored by daily palpation (>100 days) in BMT recipients that were pre-conditioned with irradiation (3 Gy, *n* = 6, open circles), Tregs (0-Gy Tregs, *n* = 5, filled squares) and unirradiated control BMT recipients (0-Gy control, *n* = 5, open triangles). (C) Clinical ISHLT rejection score of cardiac allografts >100 days after transplantation into BMT recipients treated with 3-Gy irradiation or therapeutic Treg infusion (0-Gy Tregs; *p* = 0.010, Fisher’s exact test). (D–F) Representative features of cardiac histopathology harvested >100 days after transplantation of BALB/c hearts into (D) 3-Gy–irradiated chimeras, (E) Treg-treated chimeras or (F) syngeneic recipients. Compared with syngeneic allografts, 3-Gy–irradiated chimeras show areas with increased lymphocytic infiltrates (black arrow, left) and intimal proliferation (black arrows, right) and arterial occlusion (white arrow, right). Heart allografts were fixed and stained with HE (original magnifications: ×100 [left] and ×200 [middle]) and EvG (original magnification: ×400 [right]).

To assess donor-specific tolerance, skin transplants were performed 4 to 8 weeks post-BMT. All chimeras induced by Treg treatment and the majority of chimeras pre-treated with 3-Gy TBI accepted donor skin for the length of follow-up, whereas unirradiated control mice without Tregs rejected donor skin (Figure 3A). Third-party grafts were rapidly rejected in all groups, indicating immunocompetence in these mice (data not shown). Although skin grafts are commonly considered to be the most stringent test for tolerance, primarily vascularized grafts are more suitable for studying chronic rejection. Therefore, chimeras of both groups received additional donor cardiac grafts ~2 to 4 weeks after skin grafting. Donor hearts remained viable with a palpable heart beat for the length of follow-up (100 to 160 days) in all chimeras having received either Tregs or irradiation (Figure 3B). In contrast, histopathologic examination revealed marked differences between the two groups. Treg-treated chimeras showed no or mild signs of cellular rejection (Figure 3C and D), whereas chimeras induced by irradiation showed moderate to severe signs of rejection, including lymphocyte infiltrates, destruction of cardiac muscle fibers, thickening of the intima, arteriosclerosis and fibrosis (Figure 3C and E).

Cellular rejection was significantly more frequent in chimeras induced by irradiation than in those induced by Tregs (*p* = 0.010). Fragmented elastic fibers could also be demonstrated in the myoelastic tissue of cardiac vessels of 3-Gy chimeras (but not Treg chimeras or syngeneic controls) using EvG staining. Allograft vasculopathy found in 3-Gy chimeras included interstitial hemorrhage, intravascular macrophages, capillary fragmentation, mixed inflammatory infiltrates, endothelial cell pyknosis, karyorrhexis and marked edema, indicating moderate to severe signs of chronic rejection.^19^ Histopathologic criteria for antibody-mediated rejection (AMRh), signified by endothelial swelling and pericapillar macrophages, were seen in all chimeras induced by irradiation. Notably, none of the specimens from Treg chimeras and syngeneic controls were suspicious for pathologic AMR (pAMRh). Thus, tolerance protocols relying on non-myeloablative irradition to facilitate BM engraftment seemed to induce split rather that full donor-specific tolerance in many cases.

### Donor-specific tolerance induced by Treg treatment is mainly mediated by non-deletional mechanisms

**Figure 4:**
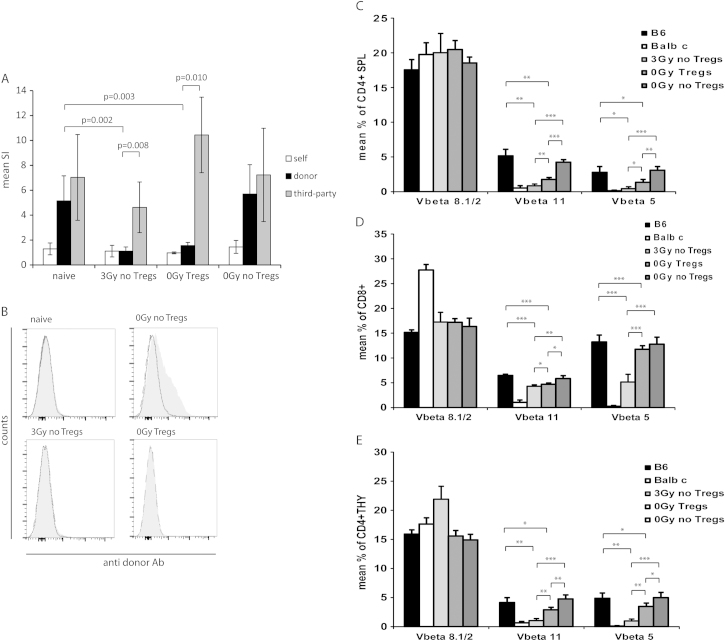
Chimeras induced by Treg treatment show donor-specific tolerance in vitro, although there is less central and peripheral deletion of donor-reactive T cells. (A) Donor reactivity was assessed in MLRs at the end of follow-up (7 months post-BMT). Chimeras induced with 3-Gy irradiation (*n* = 6) and Treg-treated BMT recipients (*n* = 4) showed specific hyporesponsiveness toward fully mismatched donor antigen in vitro compared with naive B6 mice (*n* = 7; *p* = 0.002 vs 3-Gy chimeras and *p* = 0.003 vs Treg chimeras). No donor-specific hyporesponsiveness was evident in control BMT recipients (*n* = 6) receiving BM, costimulation blockade and rapamycin only. Reactivity against third party was preserved in both groups (donor vs third-party: *p* = 0.008 for 3-Gy chimeras and *p* = 0.010 for Treg chimeras). SIs were calculated by dividing the mean count per million (cpm) from responses against recipient (open bars, B6), donor (filled bars, BALB/c) or third-party (shaded bars, C3H) stimulator cells by mean background cpm (i.e., cpm with no stimulator population). The *p-*values are shown for comparison between groups (Student’s *t*-test). Error bars indicate standard deviation. (B) Groups of mice grafted with allogeneic heart grafts were analyzed for presence of anti-donor antibodies in serum >5 months post-BMT. Chimeras induced with either irradiation (*n* = 6) or Tregs (*n* = 4) uniformly failed to develop detectable levels of anti-donor antibodies, whereas non-chimeric control mice (*n* = 5) developed substantial donor-specific antibody levels. The reactivity of sera with syngeneic (B6, dashed shaded line) and donor (BALB/c, solid gray line) thymocytes is shown by flow cytomery through indirect staining with anti-mouse IgG. Representative histograms are shown. (C) Chimeras induced by irradiation show significant deletion among donor-reactive T cells, as measured by percentages of Vβ11 and Vβ5 (but not Vβ8) CD4 T-cell splenocytes (SPL) (3-Gy chimeras vs naive B6: *p* = 0.0019 for Vβ11, *p* = 0.0081 for Vβ5; 3-Gy chimeras vs 0-Gy control: *p* < 0.0001 for Vβ11, *p* < 0.0001 for Vβ5). Deletion among Treg chimeras was evident (Treg chimeras vs naive B6: *p* = 0.0037 for Vβ11, *p* = 0.0311 for Vβ5; 0-Gy Treg chimeras vs 0-Gy Treg chimeras: *p* < 0.0001 for Vβ11, *p* = 0.0010 for Vβ5), but far less pronounced (3-Gy chimeras vs Treg chimeras: *p* = 0.0014 for Vβ11, *p* = 0.0140 for Vβ5). (D) Deletion of CD8-positive splenocytes (SPL) is shown in comparison to naive B6 controls (3-Gy chimeras: *p* < 0.0001 for Vβ11, *p* = 0.0001 for Vβ5; 0-Gy Treg chimeras: *p* = 0.0001 for Vβ11, *p* = not statistically significant [NS] for Vβ5) and in comparison to unirradiated BMT recipients without Tregs (3-Gy chimeras: *p* = 0.0016 for Vβ11, *p* < 0.0001 for Vβ5; 0-Gy Treg chimeras: *p* = 0.0077 for Vβ11, *p* = NS for Vβ5). Intrathymic deletion of CD8 cells was significantly more distinct in chimeras induced by irradiation (3 Gy vs 0-Gy Tregs: *p* = 0.0451 for Vβ11, *p* < 0.0001 for Vβ5). (E) Central deletion among single-positive CD4 thymocytes (THY) led to significantly reduced percentages of Vβ11- and Vβ5-expressing cells compared with naive B6 mice for BMT recipients receiving 3-Gy treatment (*p* = 0.0031 for Vβ11, *p* = 0.0020 for Vβ5) and BMT recipients receiving Tregs (*p* = 0.0495 for Vβ11, *p* = 0.0482 for Vβ5). Donor-reactive thymocytes in chimeras were also substantially reduced compared with control BMT recipients (3 Gy: *p* = 0.0001 for Vβ11, *p* = 0.0003 for Vβ5; 0-Gy Tregs: *p* = 0.0019 for Vβ11, *p* = 0.0204 for Vβ5), with significantly higher deletion in irradiated BMT (3-Gy vs 0-Gy Tregs: *p* = 0.0008 for Vβ11, *p* = 0.0012 for Vβ5). Deletion of donor-reactive T cells was assessed by multicolor flow cytometry in selected mice at the end of follow-up at ~7 months post-BMT (light-shaded bars: 3 Gy, *n* = 6; medium-shaded bars: 0-Gy Tregs, *n* = 4; dark-shaded bars: 0-Gy control, *n* = 5). Filled bars: naive B6 controls (*n* = 4); open bars: naive BALB/c controls (*n* = 4). ^***^p < 0.0005; ^**^p < 0.005; and ^*^p < 0.05 (Student’s *t*-test). Error bars indicate standard deviation.

To further assess tolerance among chimeras induced with irradiation or Treg treatment, in vitro MLR assays were performed. Responsiveness toward the donor was reduced almost to the level of self-reactivity in both irradiated and Treg-treated chimeras, whereas third-party reactivity was preserved (>20 weeks post-BMT) (Figure 4A). To assess tolerance at the humoral level, we tested BMT recipients for the presence of donor-specific antibodies late after BMT (>6 months). No donor-specific antobodies were detectable in 3-Gy or Treg-treated chimeras, whereas substantial levels of antibodies toward donor antibodies were demonstrated in non-chimeric control mice (Figure 4B). Substantial levels of antibodies specific for third-party antigens were detectable in chimeras upon rejection of third-party skin (data not shown). The lack of donor-specific antibodies in mice that developed signs of chronic rejection suggests humoral immunity is not the main effector mechanism mediating chronic heart graft rejection in chimeras induced by irradiation.

Clonal deletion is one of the main tolerance mechanisms in mixed chimerism models.^5^, ^20^ The frequency of superantigen-reactive T-cell populations was shown to correlate with the deletion of truly alloreactive donor-specific T cells,^21^, ^22^ thus serving as useful surrogate parameter for clonal deletion in this model. Frequencies of Vβ11^+^ and Vβ5^+^ T cells allow assessment of donor-reactive T cells in the strain combination used in the present studies (Vβ8 serves a specificity control). As reported previously,^7^ clonal deletion is less pronounced in mixed chimeras induced by therapeutic Treg infusion. We observed deletion of donor-specific T cells to be evident, but less complete, in contrast to BMT recipients given irradiation to facilitate BM engraftment, yet no deletion was seen in BMT recipients without Treg treatment.

Deletion among CD4 splenocytes was almost complete in 3-Gy chimeras late after BMT (7 months post-BMT), whereas deletion in Treg-treated chimeras was evident but significantly less substantial (Vβ11: 3 Gy 0.84% vs Tregs 1.76% [*p* = 0.0014]; Vβ5: 3 Gy 0.43% vs Tregs 1.34% [*p* = 0.0140]) (Figure 4C). Deletion among CD8 T cells, which can only be deleted intrathymically at the double-positive stage, was evident but less pronounced in both groups, indicating that central deletion occurs, but at the same time suggesting a more pronounced role for peripheral deletion (Figure 4D) (Vβ11: 3 Gy 4.30% vs Tregs 4.72% [*p* = 0.0451]; Vβ5: 3 Gy 5.16% vs Tregs 11.77% [*p* = 0.0001]) (Figure 4D). Furthermore, central deletion was directly assessed among CD4 single-positive thymocytes, again showing central deletion to be incomplete (Vβ11: 3 Gy 1.04% vs Tregs 2.87% [*p* = 0.0008]; Vβ5: 3 Gy 0.97% vs Tregs 3.47% [*p* = 0.0012]) (Figure 4E).

Thus, deletional mechanisms seem to be less important in chimeras induced by Treg treatment than in chimeras induced by non-myeloablative irradiation.

## Discussion

In this study we have demonstrated that therapeutic Treg treatment as part of a BMT protocol to induce chimerism efficiently prevents chronic rejection of murine heart allografts. Thus, additional Treg treatment not only obviates the need for cytotoxic recipient treatment (e.g., TBI), but also improves outcome after cardiac transplantation in a murine mixed chimerism model.

The mixed chimerism approach has been shown to potently induce robust donor-specific tolerance in both animals and humans.^1^ Central tolerance is the backbone of all mixed chimerism protocols, distinguishing the chimerism concept from other tolerance approaches. In addition, peripheral tolerance mechanisms lead to tolerization of the pre-existing alloreactive T-cell repertoire. The exact mechanisms and relevance of peripheral tolerance seem to differ between models, depending on the nature of the BMT protocol (e.g., myeloablation, T-cell depletion and costimulation blockade). Regulatory T cells (and other regulatory mechanisms) have been shown to be indispensable in several costimulation blockade-based mixed chimerism protocols.^6^, ^23^ In a recently reported cyclophosphamide/busulfan-based BMT protocol employing transfer of donor-derived DN Tregs, clonal deletion was again the main mechanism identified.^24^

Herein we have shown that polyclonal recipient Tregs can substitute cytotoxic recipient irradiation to facilitate BM engraftment and induce chimerism. Moreover, we demonstrated that this Treg-based protocol is superior to protocols based on irradiation in the prevention of chronic rejection of heart allografts. Notably, levels of chimerism induced with mild TBI are significantly higher than in BMT recipients receiving Treg therapy instead. However, there is no clear evidence indicating which level of chimerism is needed to induce tolerance. Besides, it is unclear whether higher levels of chimerism per se are more likely to induce full tolerance. In the clinical setting, it was shown that tolerance toward renal allografts can be achieved with transient chimerism.^2^ Also, it was found that T-cell chimerism correlates with the development of donor-specific tolerance and that high levels of hematopoietic (non–T-cell) chimerism are not necessarily associated with donor-specific tolerance.^18^, ^25^, ^26^ Importantly, T-cell tolerance was achieved with the Treg BMT protocol as demonstrated by durable CD4 and CD8 T-cell chimerism in blood and spleen, in vitro tolerance and clonal deletion of donor-reactive T cells.

We also found that the therapeutic use of polyclonal recipient Tregs in a non-cytotoxic BMT model induced full immunologic tolerance toward skin and heart allografts. Transplantation of skin/heart allografts for testing donor-specific tolerance was performed with some delay after BMT in order to avoid non-specific immunosuppressive effects that could result from the drugs used for recipient conditioning (CTLA4Ig, anti-CD154 and rapamycin). Notably, both in vitro and in vivo donor-specific tolerance are established with this regimen immediately after BMT (unpublished data from our laboratory and).^7^ Interestingly, although both skin and heart allografts remained viable for the length of follow-up, cardiac allografts from chimeras induced with a low dose of TBI developed severe signs of chronic heart rejection. In our opinion this is of clinical relevance, as often skin grafts are considered to be the most stringent tolerance test in pre-clinical models. However, these data re-emphasize that there are limitations regarding the assessment of chronic rejection when skin graft survival is used as the only end-point.

Importantly, chimeras uniformly lacked detectable levels of donor-specific antibiodies, demonstrating humoral tolerance in these mice. This is of clinical relevance as one clinical pilot trial of chimerism showed that operationally tolerant patients developed donor-specific antibodies, with some even showing complement depositions in the graft.^2^, ^27^

As mentioned previously, immunologic tolerance depends on several complementary mechanisms, including intrathymic and peripheral deletion of alloreactive T cells. In this study, we demonstrated the presence of central tolerance by intrathymic deletion as well as peripheral deletion in both BMT conditioning regimens. Notably, deletion of donor-reactive T cells in Treg-induced chimeras was less pronounced and incomplete even 7 months post-BMT, suggesting other mechanisms to be more important in this model. The presence of donor-reactive T cells, despite the superior histologic outcome of cardiac allografts, suggests that dependence of active regulatory tolerance mechanisms rather than complete clonal deletion may be favorable for induction of full tolerance. This is in contrast to studies in a graft-versus-host-disease model where the administration of double-negative Tregs was shown to mediate clonal deletion of T and natural killer cells.^24^

This new tolerance approach shows remarkable potency in the establishment of donor-specific tolerance including the prevention of chronic rejection and the potential for clinical translation. Nevertheless, implementation of Treg therapy into the clinical setting remains challenging. Although initial trials using Treg therapy in clinical hematopoietic stem cell transplantation seem promising, there are still many questions to be answered. Safety concerns include a potentially non-specific immunosuppressive effect of Treg therapy that may lead to an increased risk of infections or malignancies. In addition, lineage instability of transferred Tregs could cause them to lose their suppressor phenotype and become effector T cells eventually.^28^ Currently, trials by several study groups (e.g., the ONE study) are addressing these issues and many other questions regarding clinical practicability and the therapeutic efficiency of different Treg populations.

The use of young mice raised in a protected environment is a limitation of the present study due to avoidance of heterologous immunity and immunosenescence, which are known to impede translation of murine mixed chimerism protocols in the clinic. Strategies to overcome these hurdles have been reported, however,^29^, ^30^, ^31^ and may be applied to the present protocol. Also, as the murine heterotopic heart transplantation model has technical limitations when it comes to detection of vessel lesions by angiography or detection of complement split products (which are implemented in definitions and standards for human heart transplantation),^19^, ^32^ further studies (including NHP studies) are needed to understand the active regulatory mechanisms after Treg therapy and confirm the absence of chronic rejection.

In conclusion, we have demonstrated that Tregs can be used to enhance BM engraftment, rendering cytotoxic recipient treatment unnecessary, and can protect cardiac allografts from chronic rejection. These results underline the clinical potential of therapeutic Treg treatment in induction of BMT-based hematopoietic chimerism and subsequent prevention of acute and chronic allograft rejection. We believe these results are relevant in the development of new protocols for tolerance induction in cardiac transplantation.

## Disclosure statement

The authors have no conflicts of interest to disclose.

This work was supported by a research award from the International Society for Heart and Lung Transplantation (Research Fellowship award to N.P.) and the Austrian Science Fund (FWF TRP151-B19 to T.W.).

We thank Dr A. Zuckermann for critical reading of the manuscript.
